# Schwann Cell Expression Pattern in Human Melanomas: In Silico and Immunohistochemical Analyses

**DOI:** 10.3390/ijms27188260

**Published:** 2026-09-16

**Authors:** Claudia Giampietri, Francesca Somma, Elisa Pizzichini, Daniela D’Arcangelo, Siavash Rahimi, Cinzia Fabrizi, Antonio Facchiano

**Affiliations:** 1 Department of Anatomy, Histology, Forensic Medicine and Orthopedics, Sapienza University of Rome, 00161 Rome, Italy; claudia.giampietri@uniroma1.it (C.G.); francesca.somma@uniroma1.it (F.S.); elisa.pizzichini@uniroma1.it (E.P.); 2 Istituto Dermopatico dell’Immacolata, IDI-IRCCS, 00167 Rome, Italya.facchiano@idi.it or a.facchiano@unilink.it (A.F.); 3 Department of Life Sciences, Health and Health Professions, Link Campus University, 00165 Rome, Italy

**Keywords:** Schwann cells, melanoma, bioinformatic analysis

## Abstract

Considerable interest has recently grown toward the role Schwann cells (SCs) play in controlling the growth of several tumors. TCGA human melanoma database was interrogated focusing on 30 genes preferentially expressed in SCs and not present in melanoma (here referred to as *SC-signature*). The KM-plotter platform was exploited to address the relation of *SC-signature* to survival in melanoma patients and in other tumors as specificity controls. In addition, PCA in the GEPIA2 portal was used to address whether the *SC-signature* discriminates melanomas vs. healthy controls. Finally, the expression of one of the components of the *SC-signature* (Growth-Associated Protein 43, GAP43) was analyzed by immunohistochemistry. High levels of *SC-signature* expression were found to correlate inversely with survival in melanoma (423 patients) with strong statistical significance (HR = 2.12, *p* = 0.000003). A permutation test and validation on an independent melanoma database supported the relevance of these findings. PCA of the *SC-signature* revealed its capacity to discriminate melanomas from healthy controls. Consistently, immunohistochemical analysis revealed high expression of GAP43 in melanomas compared with control nevi. We have identified a group of 30 genes (*SC-signature*) whose expression negatively correlates with survival in melanoma patients, representing a promising prognostic biomarker candidate.

## 1. Introduction

Recent studies have highlighted the active role of Schwann cells (SCs), the main glial component of the peripheral nervous system, in promoting cancer progression and invasion [1]. In this regard, SCs influence the characteristics of the tumor microenvironment by secreting extracellular matrix-degrading enzymes and neurotrophic factors and by playing a role in immunoregulation, tumor-related pain and nerve remodeling [2]. SCs detected in cancerous tissue bear a strong resemblance to nerve repair SCs (rSCs), characterized by expression of the transcription factor c-Jun, followed by the upregulation of markers such as neurotrophin receptor p75 NTR (NGFR), glial fibrillary acidic protein (GFAP), Growth-Associated Protein 43 (GAP43), and Sox-10 [2,3,4,5,6]. S100B is detected in both uninjured peripheral nerves and in activated/repair SCs [7]. Also in melanomas, SCs were shown to infiltrate the tumor tissue and undergo reactive dedifferentiation resembling that observed after nerve injury [5]. In this regard, a remarkable study has shown that the genetic ablation of SCs led to a slowdown in melanoma growth in mouse models, whilst in human melanoma patients, high expression of some genes related to SCs correlated with worse prognosis [8].

Our data extend and reinforce those previous findings, demonstrating that a set of 30 genes expressed by SCs (hereinafter referred to as the *SC-signature*) inversely correlates with survival in melanoma patients. In line with the in silico analysis, tissue sections show strong staining for the rSC marker GAP43 (Growth-Associated Protein 43) in the peritumoral area of human melanomas, whilst no such staining is observed around melanocytic nests of control nevi.

These results support the possible function of certain components of the peripheral nervous system in governing melanoma growth and shed new light on SC markers for possible prognostic and diagnostic applications.

## 2. Results

### 2.1. Selection of Genes for the SC-Signature

A non-automated literature search enabled the selection of genes with the following characteristics: (i) preferential expression in SCs according to PubMed [9,10,11,12,13,14,15,16,17,18,19,20,21,22,23,24,25,26,27,28,29,30,31,32]; (ii) undetectable protein expression in melanoma cells as reported in the Human Protein Atlas (https://www.proteinatlas.org/). Based on these selection criteria, 30 genes were identified that are preferentially expressed by SCs but have not been observed in melanoma cells. Table 1 reports the selected genes, their main known biological role and the corresponding reference. The selected 30 genes (here referred to as “*SC-signature*”) should not be considered an exhaustive overview of the genes expressed by SCs. In line with our aims, it is, rather, a useful tool for investigating the biological properties of the SCs in melanoma.

**Table 1:** Genes of the *SC-signature*, listed in alphabetical order.

	Symbol	Role (According to Genecards at https://www.genecards.org/)	Role in SCs	Ref.
1	*ADGRG6*	G protein-coupled receptor	Development of myelinating SCs	[9]
2	*AQP1*	Water channel protein	Water homeostasis in SCs	[10]
3	*B3GAT1*	Member of the glucuronyl transferase gene family (*CD57* or *LEU7*)	Marker associated with SC maturation and myelination	[11]
4	*BDNF*	Member of the nerve growth factor family	Marker of rSCs	[12]
5	*CADM4*	Cell adhesion molecule	Cell adhesion molecule expressed in myelinating SCs (it mediates SC–axon adhesion)	[13]
6	*CDH19*	Type II cadherin, cell–cell adhesion	Marker of SC precursors during development	[14]
7	*CNP*	2′,3′-cyclic-nucleotide 3′-phosphodiesterase activity	Myelin-associated enzyme in SCs	[15]
8	*CNTF*	Promotes neurotransmitter synthesis and neurite outgrowth	Expressed in myelinating or promyelinating SCs that are in contact with axons	[16]
9	*CUEDC2*	Enables ubiquitin binding activity	Marker of SCs in skin biopsy samples	[17]
10	*DRP2*	Members of the dystrophin family	In myelinating SCs for proper myelin sheath formation	[18]
11	*EDNRB*	G protein-coupled receptor	Myelination regulator	[19]
12	*ERBB2*	Member of the epidermal growth factor (EGF) receptor	rSCs	[20]
13	*FYN*	Member of the protein-tyrosine kinase oncogene family	SC migration	[21]
14	*GALC*	Hydrolyzes the galactose ester bonds in sphingolipids	Myelinating SCs	[22]
15	*GAP43*	Crucial component of an effective regenerative response in the nervous system	Non-myelin-forming SCs and rSCs	[23]
16	*GDNF*	Encodes a secreted ligand of TGF-beta superfamily of proteins	Non-myelinating and rSCs	[17]
17	*GFAP*	Major intermediate filament proteins of mature astrocytes	Remak and rSCs	[24]
18	*GPM6B*	Membrane glycoprotein involved in cellular housekeeping functions	Myelinating SCs	[25]
19	*ITGB4*	Integrin Subunit Beta 4	Myelinating SCs	[26]
20	*KCNJ10*	Member of the inward rectifier-type potassium channel family (*Kir4.1*)	Non-myelinating SCs	[27]
21	*LUM*	It may regulate collagen fibril organization	Modified SCs of Pacinian corpuscles	[28]
22	*MAG*	Myelin Associated Glycoprotein	Myelinating SCs	[17]
23	*MBP*	Myelin Basic Protein, major constituent of the myelin	Myelinating SCs	[17]
24	*PLLP*	Plasmolipin, involved in myelin assembly and regulation of transcytosis	Myelinating SCs	[17]
25	*PLP1*	Proteolipid, predominant component of myelin	Myelinating SCs	[17]
26	*RBPJ*	Transcriptional regulator of the Notch signaling pathway	SC precursors (SCPs) (early neural crest–derived glial precursors) and immature SCs	[29]
27	*RXRG*	Retinoid X Receptor Gamma	rSCs	[30]
28	*SCN7A*	Sodium Voltage-Gated Channel Alpha Subunit 7	Non-myelinating SCs	[31]
29	*SLC6A2*	Member of the sodium: neurotransmitter symporter family	Non-myelinating SCs	[31]
30	*VGLL3*	Involved in regulation of transcription by RNA polymerase II	Schwannoma cells (neoplastic SC-like)	[32]

### 2.2. SC-Signature and Survival Analysis in Melanoma Patients

**Figure 1:**
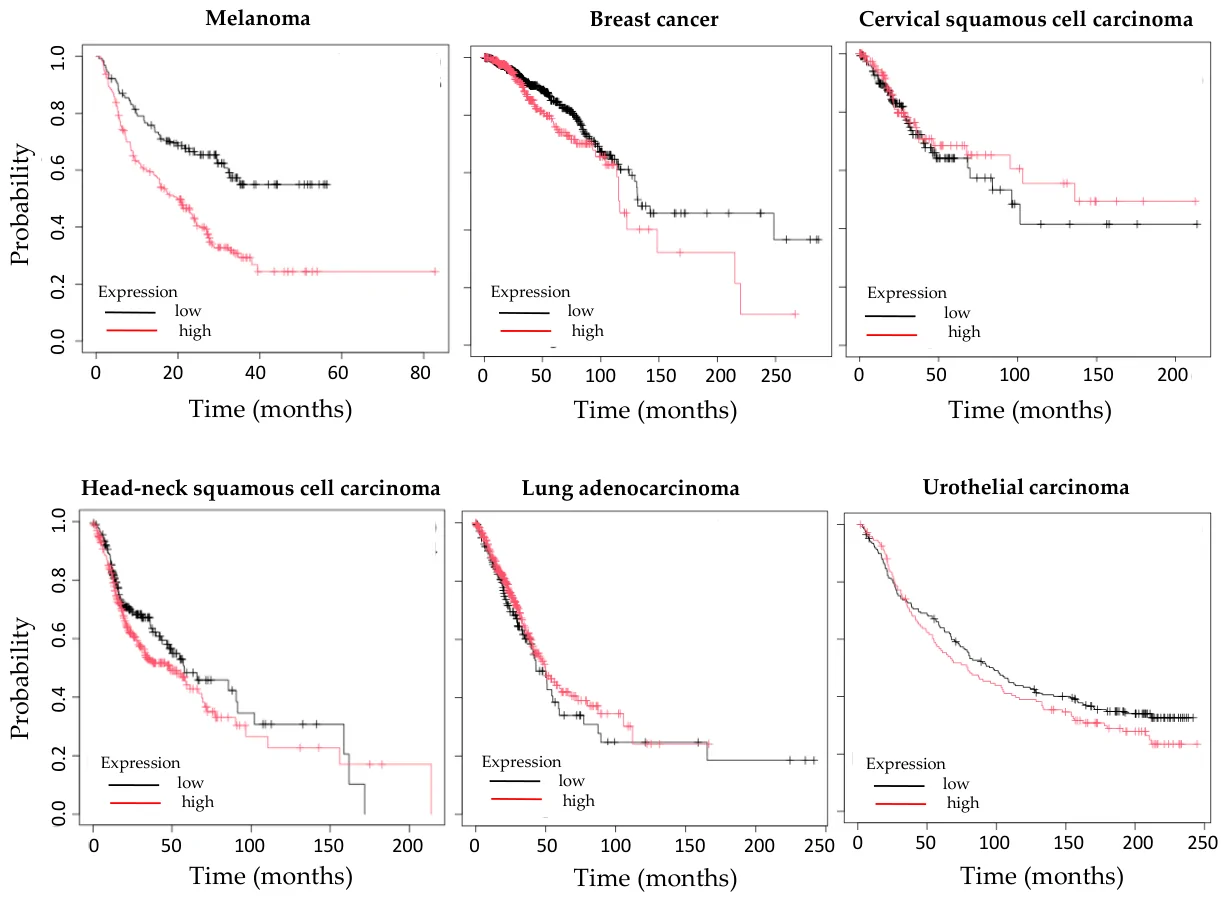
*SC-signature* association with survival probability in melanoma patients and in five other cancers (TCGA database). Only in melanoma patients, high expression of the 30 genes of the *SC-signature* associates with a significantly reduced probability of survival according to the KM plotter portal. High *SC-signature* expression (red line); low *SC-signature* expression (black line). The time component (months), shown on the *x*-axis, varies depending on the time of follow up in each tumor cohort.

Genes reported in Table 1 were analyzed as a “profile” in the Kaplan–Meier (KM) plotter portal to investigate whether their combined expression associates with overall survival in human melanomas. Figure 1 indicates how the expression of the *SC-signature* correlates with survival in melanoma and in five other cancers (breast cancer, cervical squamous cell carcinoma, head-neck squamous cell carcinoma, lung adenocarcinoma, urothelial carcinoma), on The Cancer Genome Atlas (TCGA) datasets, according to the KM Plotter portal. The melanoma dataset is the only dataset showing a significant association of *SC-signature* with survival. Table 2 reports the statistical parameters corresponding to each tumor investigated. Namely, high *SC-signature* expression significantly associates with worse prognosis (HR = 2.12, logrank *p* = 3 × 10^−6^). The melanoma dataset is also the only dataset showing a false discovery rate ≤ 1% (FDR ≤ 1%), further indicating strong statistical significance.

To further support the relevance of the *SC-signature* in melanoma, a permutation test was carried out to assess how the association of the given signature differs from the association of a set of randomly chosen ones. To this aim, 100 profiles of 30 randomly chosen genes were analyzed under conditions identical to those used to analyze the *SC-signature*. The permutation analysis indicated that the association of the *SC-signature* with survival in melanoma patients is significantly different from that of the randomized profiles (permutation *p* = 0.01) (see Supplementary Table S1).

**Figure 2:**
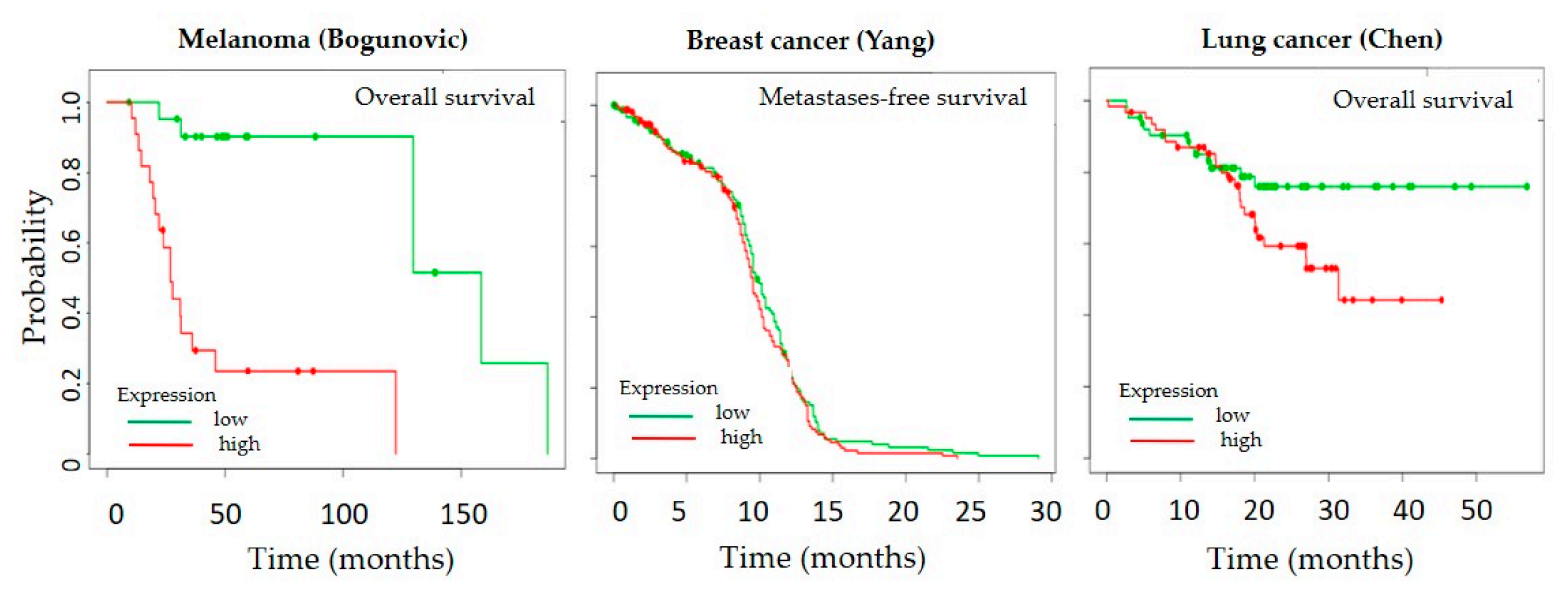
*SC-signature* association with survival probability in melanoma, breast and lung cancers (GSE datasets; SurvExpress platform). High expression of the *SC-signature* (red) associates with a significantly reduced survival probability in melanoma patients, while in breast and lung cancer datasets no such association is observed according to the SurvExpress platform. High *SC-signature* expression (red line); low *SC-signature* expression (green line). The time component (months) shown on the *x*-axis varies depending on the time of follow up in each tumor cohort.

The statistical relevance of the 30 genes of the *SC-signature* was additionally validated in independent databases via the online platform SurvExpress at http://victortrevino.bioinformatics.mx:8080/Biomatec/SurvivaX.jsp (accessed on 30 June 2026). The corresponding KM curves are reported in Figure 2. Namely, the effect of the *SC-signature* on survival of melanoma patients was validated on the Bogunovic Skin Survival GSE19234, reporting data from 44 patients. On the other hand, the *SC-signature* shows no significant association in a breast cancer dataset (the Yang Breast GSE113863 with 304 patients), and in a lung cancer dataset (the Chen Lung GSE4882, with 125 patients). Table 2 reports the statistical parameters corresponding to each tumor investigated. This validation analysis confirms in independent databases the strong association shown in Figure 1 between high survival rates in melanoma patients and low expression of the *SC-signature*. Furthermore, this association appears again to be specific to melanoma, as it is not found in breast and lung cancer (Figure 2).

### 2.3. Principal Component Analysis (PCA) in Melanoma

**Figure 3:**
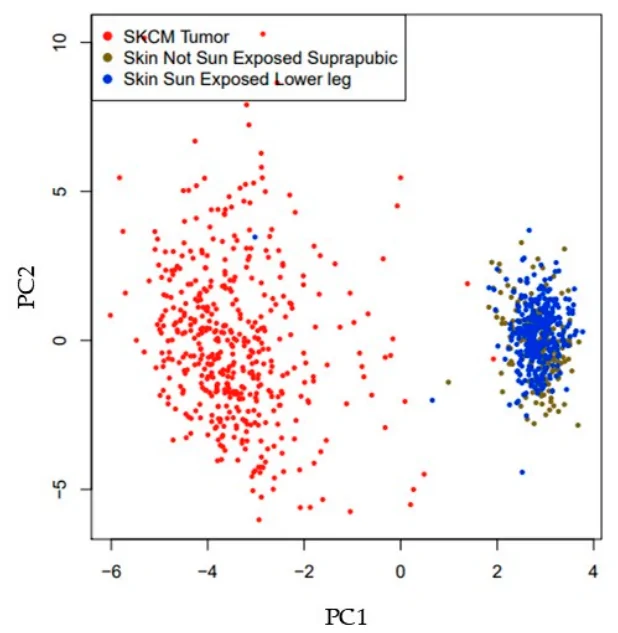
Principal Component Analysis (PCA) of the *SC-signature* expression in melanoma patients and in healthy controls. The PCA carried out on the expression of the *SC-signature* clearly differentiates melanoma cases (red dots) from healthy controls (blue and green dots), corresponding to biopsies from sun-exposed (blue dots) and sun-unexposed healthy skin (green dots). This analysis indicates that genes of the *SC-signature* are differently expressed in melanoma vs. healthy controls.

The *SC-signature* was then tested in a dimensionality reduction tool (PCA) in the GEPIA2 (Gene Expression Profiling Interactive Analysis 2) portal to assess a potential diagnostic role in melanoma patients. Figure 3 shows the 2D scatter plot of the two main components, according to this analysis. It indicates that the *SC-signature* is capable of clearly distinguishing melanoma samples (red dots) from the corresponding healthy skin controls. As highlighted in the figure, identical results are obtained whether the control group consists of skin exposed to the sun (blue dots) or skin not exposed to the sun (green dots).

Altogether, the results shown above strongly suggest a specific role of SC-expressed genes in melanoma and raise the hypothesis that the identified *SC-signature* might have distinct biological implications in melanoma patients.

### 2.4. GAP43 Detection in Melanoma by Immunohistochemistry

**Figure 4:**
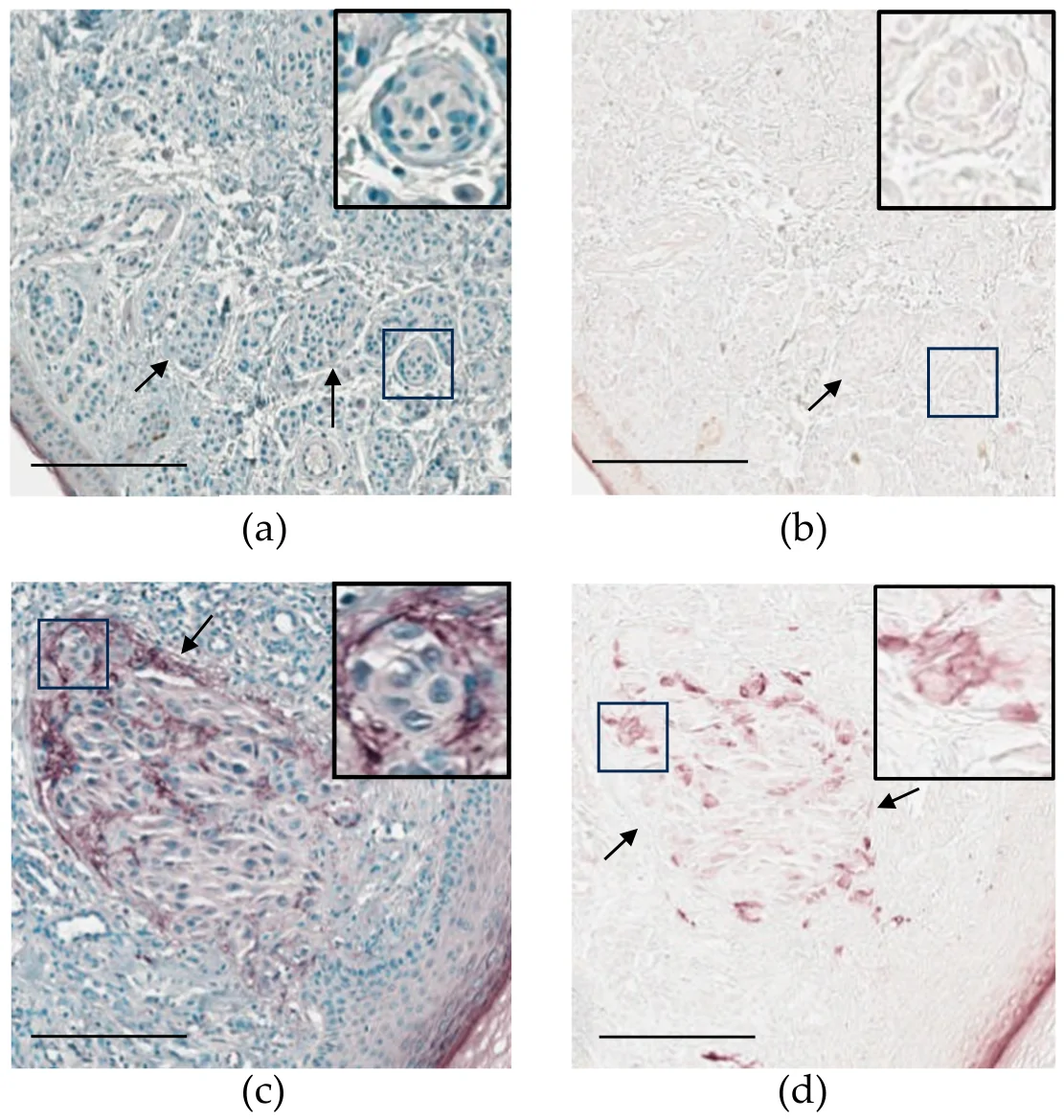
Representative images showing the differential expression of GAP43 and p75NTR in human melanoma vs. melanocytic control nevus. Purple staining (VIP) indicates positivity. (**a**) Control nevus, GAP43 staining is negative on the edges of melanocytic cell nests (arrows) and inside melanocytic cells (detail is shown in the enlarged insert at the top right). (**b**) Control nevus, p75NTR staining is negative both around melanocytic cell nests (arrow) and in melanocytic cells (insert at higher magnification). (**c**) Melanoma, GAP43 staining is observed in the peritumoral area around melanoma cell nests (arrow) but is absent in the melanoma cells (see the enlarged insert at the top right-hand corner). (**d**) Melanoma, p75NTR staining is lacking in the peritumoral area (arrows) yet is visible in some melanoma cells (see the insert at higher magnification). Nuclei are counterstained with methyl-green in (**a**,**c**) to highlight the morphological feature of the samples. Scale bars: 200 μm.

Next, we analyzed by immunohistochemistry the expression and localization of one of the components of the *SC-signature* (Growth Associated Protein 43, GAP43), known to be expressed in non-myelin-forming SCs and rSCs [23]. As shown in Figure 4, in tissue sections from human melanoma biopsies GAP43+ cells appear mainly associated with the peritumoral area. In the higher-magnification view, note that GAP43 positivity is confined to the peritumoral area, whilst melanoma cells are unmarked (Figure 4c). We also analyzed by immunohistochemistry one of the most common SC markers, p75NTR (neurotrophin receptor p75 NTR) that has been excluded from our *SC-signature* as it has been previously reported to be expressed by some melanomas. Indeed, as shown in the figure (Figure 4d), p75NTR marks some melanoma cells in line with the data provided by the Human Protein Atlas (https://www.proteinatlas.org/). In control melanocytic nevi, no positive staining was observed for either GAP43 or p75NTR (Figure 4a,b).

The immunohistochemical data on GAP43 expression in melanomas compared with control nevi are therefore consistent with those obtained in silico.

## 3. Discussion

SCs are not only supportive elements for peripheral nerves since they actively regulate the tumor microenvironment, promoting or inhibiting neoplasm growth. Rather than being passive bystanders, SCs are known to contribute to tumor progression by activating developmental and regenerative programs that are usually triggered during nerve repair [33,34]. In fact, in the tumor microenvironment, SCs undergo rSCs-like reprogramming, acquiring enhanced mobility and sustaining immunosuppressive signals [35]. Zhang and colleagues highlight the role of SCs in modulating Natural Killer (NK) cells [36]. In addition, SCs release cytokines that create an immunosuppressive environment, facilitating tumor progression and perineural invasion. Deborde and Wong [3] show that SCs produce growth factors that nourish tumor cells and facilitate metastasis formation along nerve bundles. In more detail, melanoma-activated SCs show a repair phenotype which may aid tumor growth through SC interaction with melanoma cells and the facilitation of an immunosuppressive microenvironment [5]. Thus, peripheral nerves and their associated SCs form a highly dynamic and functional environment that melanoma cells can exploit.

On the whole, these studies converge to demonstrate that SCs are not passive bystanders. Under tumor-derived stimulation, they release chemokines and neurotrophic factors that increase the aggressiveness of malignant cells and inhibit the host’s immune response. In melanoma, SCs can contribute to creating a protective niche for melanoma cells, thereby promoting their growth. In fact, melanoma cells often migrate along nerves by exploiting the SCs themselves [5]. Furthermore, it is noteworthy that, as suggested in a preprint study [37], melanoma cells—which originate from melanocytes (neural crest cells)—can shift to a SC-like state (known as a “transitional phenotype”) that can foster invasiveness, resistance to therapies, and metastatic spread.

Previous work by Rocha and colleagues through in silico analysis of human tumor biopsies showed that high expression of some SC-related genes (namely, GFAP and PLP1) in melanoma shows a tendency toward worse overall survival [8]. The present study builds on this compelling finding, and indeed both GFAP and PLP1 are included in our *SC-signature*. The selection criterion we used led us to identify 30 genes that are expressed by SCs but are absent in melanoma cells. In a condition where SC and melanoma may share some expression patterns due to their common origin from the neural crest during development, such a selection procedure aimed at identifying markers as selective as possible for SCs. Although we are aware that this might not be an exhaustive list of differentially expressed genes, still it represents a large profile investigating overall survival of melanoma patients. In fact, we showed that the expression of such a 30-gene *SC-signature* is negatively associated with melanoma patient survival based on TCGA expression data, while it shows no significant association with survival in other cancers. To further assess the specificity of our findings, the permutation test showed a significantly different (*p* < 0.01) association of the *SC-signature* compared with that of 100 randomly chosen profiles, supporting the specificity and biological relevance of *SC-signature*. While it is known that a multi-gene signature captures tumor biology more comprehensively than a single-gene signature, including as many as 30 genes may introduce specific mathematical challenges [38]. To mitigate the risk of overfitting, the survival effect observed in the TCGA results was further validated in an independent melanoma dataset. Overall, our data indicate that high expression of *SC-signature* is associated with poor survival in patients with melanoma but not in patients with other cancers such as breast, cervical, lung, urothelial, head-and-neck cancers. Such data point to the fact that SCs may support melanoma growth, in line with the findings reported by other authors [5], and suggest a possible prognostic role of the identified *SC-signature*. Moreover, the in silico analyses are strengthened by the immunohistochemical data detecting GAP43 in the peritumoral area of melanoma cell nests but not in the corresponding control nevi. Even though the presence of GAP43 in the peritumoral area is consistent with the in silico analysis, we nevertheless need to bear in mind that the immunohistochemical analysis was carried out on a limited number of samples (15 melanomas and 16 nevi). Furthermore, GAP43 labeling alone is not sufficient to identify SCs. Therefore, further studies are needed to expand and consolidate these findings.

Analysis of laser-microdissected tissues offers high resolution at the cellular level, but these samples are not available in the TCGA and GSE datasets. Thus, bulk tumor datasets may show signals from malignant cells and from the surrounding stroma, which usually makes precise cell-type attribution challenging. Within the scope of our study, this may represent only a partial limitation, or rather somehow an advantage, since we are investigating the role of the surrounding stroma (including SCs) rather than the tumor itself. The 30-gene *SC-signature* includes genes expressed in SCs but not in melanoma cells; therefore, changes in bulk datasets may indeed reflect the contribution of the stroma surrounding the tumor tissue itself, at least to a reasonable extent.

Taken together, these first findings of ours, although primarily descriptive, suggest the importance of conducting a more in-depth study into the potential role of SCs in regulating the growth of melanoma. Studying melanoma-associated SCs and identifying, wherever possible, novel markers specific to tumor-associated SCs may improve early diagnosis by distinguishing healthy SCs from those reprogrammed by the tumor. In addition, these studies may pave the way for new potential therapeutic strategies that interfere with the biochemical signals (e.g., cytokines) that SCs transmit to tumor cells and vice versa.

## 4. Materials and Methods

### 4.1. SC-Signature Identification

Based on a thorough examination of manually collected data from PubMed, genes that are preferentially expressed in SCs (both myelinating and non-myelinating) were selected. Subsequently, all those that had been described in human melanoma cells, such as p75NTR, were excluded. This was carried out using data reported in the Human Protein Atlas (https://www.proteinatlas.org/).

### 4.2. In Silico Analysis

The genes listed in Table 1 (*SC-signature*) were imported into the “Immunotherapy” section of the KM plotter platform, at https://kmplot.com/analysis/index.php?p=service&cancer=immunotherapy (accessed on 30 June 2026).

Their combined expression was analyzed in relation to the survival of patients with melanoma. This was achieved via the “use multiple genes” function, then the “use mean expression of selected genes” function, and finally by restricting the analysis to the “melanoma” cohort (423 individuals). Default settings were applied to all other parameters. The probes used for the survival analysis were those suggested by KM plotter according to the gene name. All other cancer cohorts were investigated using the “mRNA pan-cancer” button at https://kmplot.com/analysis/ [39,40,41].

Validation of the SC-signature was further performed in independent databases via the online platform SurvExpress at http://victortrevino.bioinformatics.mx:8080/Biomatec/SurvivaX.jsp.

### 4.3. Statistical Analyses

To assess whether the logrank *p* of *SC-signature* association with overall survival differed significantly from what would be expected by chance, a permutation test was carried out. One hundred randomly chosen signatures composed of thirty randomly chosen genes were analyzed, and the logrank *p* was calculated for each permutation (see the exact procedure summarized below in the Randomization test paragraph), generating a null distribution of values. The observed logrank *p* was then compared against this null distribution. Statistical significance was assessed and the permutation *p* was calculated as the fraction:

*p*_perm_ = 1 + number of permutations with *p* ≤ *p* obs/permutations number + 1.

The genes composing the 100 profiles are reported in Supplementary Table S1, along with the computed HR and *p* values.

### 4.4. Randomization Test

A randomization test was carried out on 100 randomly chosen profiles vs. the *SC-signature*. The aim was to assess whether the association of the *SC-signature* with survival was significantly different to the association of a set of randomly chosen signatures, considering the logrank *p*; the randomly chosen profiles were analyzed under conditions identical to those used for the 30 genes of the *SC-signature*. The overall survival associated with each profile (the *SC-signature* and the 100 randomly chosen profiles) was investigated in the KM plotter portal, which returned the corresponding Hazard Ratio (HR) and logrank *p* value.

The permutation *p* value was obtained according to the following calculation:

Given that the SC-signature shows a survival analysis with a logrank *p* of 3 × 10^−6^ out of the 100 permutations (N) carried out, 0 (x) random profiles gave a logrank *p* < 3 × 10^−6^. Therefore, the calculation is: (1 + x)/(n + 1), i.e., (1 + 0)/100 + 1 = 0.0099, indicating computed permutation *p* = 0.01.

### 4.5. PCA Applied to the SC-Signature

The gene expression value of the *SC-signature* was then subject to Principal Component Analysis (PCA) to investigate whether this gene list can effectively differentiate melanomas from healthy controls. To this aim, the “dimensionality reduction” tool in the GEPIA2 was used, at the link http://gepia2.cancer-pku.cn/#dimension (accessed on 30 June 2026).

### 4.6. Immunohistochemistry on Human Melanoma and Benign Nevi Samples

For this retrospective pilot study, paraffin-embedded samples from the histotheca of the IDI-IRCCS were used; the presence of SC markers was assessed in 15 melanomas and 16 control samples (benign nevi). Control samples were intradermic melanocytic nevi; melanoma samples were superficial spreading melanomas infiltrating the dermis. Specimens were fixed in 10% buffered formalin for 2–4 h and embedded in low-fusion-temperature paraffin (55–57 °C), and 5 μm sections were obtained. The sections were deparaffinized in xylene and rehydrated in a decreasing ethanol series. Endogenous peroxidase activity was quenched by incubating sections in 0.45% hydrogen peroxide in absolute methanol. Heat-induced antigen retrieval was performed using sodium-citrate pH 6.0 buffer (GAP43) or TRIS/EDTA pH 9.0 (p75NTR). Non-specific binding was blocked with 3% non-fat milk in PBS. Sections were incubated overnight at +4 °C with anti-GAP43 primary antibody (Abcam, Cambridge, UK, ab75810, 1:1000) and p75NTR (Abcam, ab52987, 1:200). Then, after three washes, sections were incubated with secondary anti-rabbit biotinylated antibody (Vector Laboratories, Newark, CA, USA, BA-1000, 1:200) and treated with ABC reagent (Vector Laboratories, PK-6100). Signal was developed using VIP substrate (Vector Laboratories, SK-4600), and sections were counterstained with methyl-green (Vector Laboratories, H-3402). Thereafter, the slides were dehydrated quickly through 95% and 100% ethanol, cleared in xylene and permanently mounted. All stained slides were scanned by a digital scanner (Aperio Scanscope CS and FL System, Aperio Digital Pathology, Leica Biosystems, Milan, Italy) and processed by Aperio ImageScope software (v.12.4.6.5003).

For each sample, a digital image of the entire section was obtained using the digital scanner, but no quantification was carried out. Counterstaining with methyl-green was used to identify the peritumoral area.

### 4.7. Ethics Committee

This study was approved by Istituto Dermopatico dell’Immacolata Research Ethics Committee (Comitato etico territoriale Lazio Area 5 Prot. N. 31 CE/2022, approved on 15 July 2022).

## Figures and Tables

**Table 2:** Statistics of survival analyses.

**TCGA Dataset of** **Figure 1**					
**Cancer Type**	**N. of Patients**	**Hazard Ratio (HR)**	**Confidence Interval (C.I.)**	**Log-Rank** ***p***	**False ** **Discovery Rate (FDR)**
Melanoma	423	2.12	1.54–2.93	0.000003	1%
Breast cancer	1090	1.39	1–1.93	0.047	50%
Cervical squamous cell carcinoma	304	0.85	0.53–1.63	0.497	100%
Head-neck squamous cell carcinoma	500	1.23	0.93–1.62	0.14	100%
Lung adenocarcinoma	513	0.86	0.63–1.17	0.33	100%
Urothelial carcinoma	348	1.18	0.91–1.52	0.22	100%
**GSE Datasets of** **Figure 2**					
**Cancer Type**	**N. of Patients**	**Hazard Ratio (HR)**	**Confidence Interval (C.I.)**	**Log-Rank** ***p***	
Melanoma (Bogunovic)	44	15.76	3.55–69.93	0.000002	
Breast cancer (Yang)	304	1.13	0.88–1.44	0.325	
Lung cancer (Chen)	125	1.8	0.92–3.52	0.086	

## Data Availability

The original contributions presented in this study are included in the article/Supplementary Material. Further inquiries can be directed to the corresponding author.

## Supplementary Materials

The following supporting information can be downloaded at: https://www.mdpi.com/article/10.3390/ijms27188260/s1.

## References

[1] Ziwan H., Furui L., Lin L., Zhihui H., Yongjie W. (2025). Interplay between Schwann Cells and peripheral cancers: Mechanisms and therapeutic targets in cancer progression. Glia.

[2] Deborde S., Omelchenko T., Lyubchik A., Zhou Y., He S., McNamara W.F., Chernichenko N., Lee S.-Y., Barajas F., Chen C.-H. (2016). Schwann cells induce cancer cell dispersion and invasion. J. Clin. Investig..

[3] Deborde S., Wong R.J. (2022). The role of Schwann Cells in cancer. Adv. Biol..

[4] Demir I.E., Boldis A., Pfitzinger P.L., Teller S., Brunner E., Klose N., Kehl T., Maak M., Lesina M., Laschinger M. (2014). Investigation of Schwann cells at neoplastic cell sites before the onset of cancer invasion. JNCI J. Natl. Cancer Inst..

[5] Shurin G.V., Kruglov O., Ding F., Lin Y., Hao X., Keskinov A.A., You Z., Lokshin A.E., La Framboise W.A., Falo L.D. (2019). Melanoma-induced reprogramming of Schwann cell signaling aids tumor growth. Cancer Res..

[6] Wilcox M.B., Laranjeira S.G., Eriksson T.M., Jessen K.R., Mirsky R., Quick T.J., Phillips J.B. (2020). Characterising cellular and molecular features of human peripheral nerve. Acta Neuropathol. Commun..

[7] Sorci G., Riuzzi F., Arcuri C., Tubaro C., Bianchi R., Giambanco I., Donato R. (2013). S100B protein in tissue development, repair and regeneration. World J. Biol. Chem..

[8] Rocha B.G.S., Picoli C.C., Gonçalves B.O.P., Silva W.N., Costa A.C., Moraes M.M., Costa P.A.C., Santos G.S.P., Almeida M.R., Silva L.M. (2023). Tissue-resident glial cells associate with tumoral vasculature and promote cancer progression. Angiogenesis.

[9] Bradley E.C., Cunningham R.L., Wilde C., Morgan R.K., Klug E.A., Letcher S.M., Schöneberg T., Monk K.R., Liebscher I., Petersen S.C. (2019). In vivo identification of small molecules mediating Gpr126/Adgrg6 signaling during Schwann cell development. Ann. N. Y. Acad. Sci..

[10] Zhang J., Xiong Y., Lu L.X., Wang H., Zhang Y.F., Fang F., Song Y.L., Jiang H. (2013). AQP1 expression alterations affect morphology and water transport in Schwann cells and hypoxia-induced up-regulation of AQP1 occurs in a HIF-1alpha-dependent manner. Neuroscience.

[11] Schwechheimer K., Gass P., Berlet H.H. (1992). Expression of oligodendroglia and Schwann cell markers in human nervous system tumors: An immunomorphological study and western blot analysis. Acta Neuropathol..

[12] Yi S., Yuan Y., Chen Q., Wang X., Gong L., Liu J., Gu X., Li S. (2016). Regulation of Schwann cell proliferation and migration by miR-1 targeting Brain-Derived Neu-rotrophic Factor after peripheral nerve injury. Sci. Rep..

[13] Meng X., Maurel P., Lam I., Heffernan C., Stiffler M.A., McBeath G., Salzer J.L. (2019). Necl-4/Cadm4 recruits Par-3 to the Schwann cell adaxonal membrane. Glia.

[14] Takahashi M., Osumi N. (2005). Identification of a novel type II classical cadherin: Rat cadherin 19 is expressed in the cranial ganglia and Schwann cell precursors during development. Dev. Dyn..

[15] Sprinkle T.J. (1989). 2′,3′-cyclic nucleotide 3′-phosphodiesterase, an oligodendrocyte-Schwann cell and myelin-associated enzyme of the nervous system. Crit. Rev. Neurobiol..

[16] Lee D.A., Zurawel R.H., Windebank A.J. (1995). Ciliary neurotrophic factor expression in Schwann cells is induced by axonal contact. J. Neurochem..

[17] Svaren J., Moran J.J., Wu X., Zuccarino R., Bacon C., Bai Y., Ramesh R., Gutmann L., Anderson D.M., Pavelec D. (2019). Schwann cell transcript biomarkers for hereditary neuropathy skin biopsies. Ann. Neurol..

[18] Sherman D.L., Fabrizi C., Gillespie C.S., Brophy P.J. (2001). Specific disruption of a Schwann cell dystrophin-related protein complex in a demyelinating neuropathy. Neuron.

[19] Brinkmann B.G., Quintes S. (2017). Zeb2: Inhibiting the inhibitors in Schwann cells. Neurogenesis.

[20] Han S.B., Kim H., Lee H., Grove M., Smith G.M., Son Y.J. (2017). Postinjury induction of activated ErbB2 selectively hyperactivates denervated Schwann cells and promotes robust dorsal root axon regeneration. J. Neurosci..

[21] Paratcha G., Ledda F., Ibáñez C.F. (2003). The neural cell adhesion molecule NCAM is an alternative signaling receptor for GDNF family ligands. Cell.

[22] Stoffel W., Bosio A. (1997). Myelin glycolipids and their functions. Curr. Opin. Neurobiol..

[23] Liu Z., Jin Y.-Q., Chen L., Wang Y., Yang X., Cheng J., Wu W., Qi Z., Shen Z. (2015). Specific marker expression and cell state of Schwann Cells during culture in vitro. PLoS ONE.

[24] Triolo D., Dina G., Lorenzetti I., Malaguti M.C., Morana P., Del Carro U., Comi G., Messing A., Quattrini A., Previtali S.C. (2006). Loss of Glial Fibrillary Acidic Protein (GFAP) impairs Schwann cell proliferation and delays nerve regeneration after damage. J. Cell Sci..

[25] Bang M.L., Vainshtein A., Yang H.J., Eshed-Eisenbach Y., Devaux J., Werner H.B., Peles E. (2018). Glial M6B stabilizes the axonal membrane at peripheral nodes of Ranvier. Glia.

[26] Van der Zee C.E., Kreft M., Beckers G., Kuipers A., Sonnenberg A. (2008). Conditional deletion of the Itgb4 integrin gene in Schwann cells leads to delayed peripheral nerve regeneration. J. Neurosci..

[27] Procacci N.M., Hastings R.L., Aziz A.A., Christiansen N.M., Zhao J., De Angeli C., LeBlanc N., Notterpek L., Valdez G., Gould T.W. (2023). Kir4.1 is specifically expressed and active in non-myelinating Schwann cells. Glia.

[28] García-Piqueras J., García-Mesa Y., Feito J., García B., Quiros L.M., Martín Biedma B., Cobo T., Vega J.A., García-Suárez O. (2019). Class I and Class II small leucine- rich proteoglycans in human cutaneous pacinian corpuscles. Ann. Anat..

[29] Miller S.R., Benito C., Mirsky R., Jessen K.R., Baker C.V.H. (2018). Neural crest Notch/Rbpj signaling regulates olfactory gliogenesis and neuronal migration. Genesis.

[30] Lovatt D., Tamburino A., Krasowska-Zoladek A., Sanoja R., Li L., Peterson V., Wang X., Uslaner J. (2022). scRNA-seq generates a molecular map of emerging cell subtypes after sciatic nerve injury in rats. Commun. Biol..

[31] Pascual G., Domínguez D., Elosúa-Bayes M., Beckedorff F., Laudanna C., Bigas C., Douillet D., Greco C., Symeonidi A., Hernández I. (2021). Dietary palmitic acid promotes a prometastatic memory via Schwann Cells. Nature.

[32] Schmid S., Mirchia K., Tietze A., Liu I., Siewert C., Nückles J., Schittenhelm J., Behling F., Snuderl M., Hartmann C. (2025). VGLL fusions define a new class of intraparenchymal central nervous system schwannoma. Neuro-Oncology.

[33] Giampietri C., Pizzichini E., Somma F., Petrungaro S., De Santis E., Rahimi S., Facchiano A., Fabrizi C. (2025). Roles of peripheral nerves in tumor initiation and progression. Int. J. Mol. Sci..

[34] de Franchis V., Petrungaro S., Pizzichini E., Camerini S., Casella M., Somma F., Mandolini E., Carpino G., Overi D., Cardinale V. (2024). Cholangiocarcinoma malignant traits are promoted by Schwann cells through TGFβ signaling in a model of perineural invasion. Cells.

[35] Yang T., Zhao W.W., Zhang X., Zhang W.J., Zou R. (2026). Schwann cells as immunomodulators in the tumor immune microenvironment: Mechanisms and therapeutic implications. Life Sci..

[36] Zhang S., Chen J., Cheng F., Zheng F. (2024). The emerging role of Schwann Cells in the tumor immune microenvironment and its potential clinical application. Int. J. Mol. Sci..

[37] Gopalan V., Wong C.W., Leshem R., Owen L., Vallius T., Shi Y., Jiang Y., Pérez-Guijarro E., Wu E., Chin S. (2025). Transitory Schwann cell precursor and hybrid states underpin melanoma therapy resistance and metastasis. bioRxiv.

[38] Chang Y.-H., Chen C.-M., Chen H.-Y., Yang P.-C. (2015). Pathway-Based Gene Signatures Predicting Clinical Outcome of Lung Adenocarcinoma. Sci. Rep..

[39] D’Arcangelo D., Scatozza F., Giampietri C., Marchetti P., Facchiano F., Facchiano A. (2019). Ion channel expression in human melanoma samples: In silico identification and experimental validation of molecular targets. Cancers.

[40] Scatozza F., Facchiano A. (2022). Expression of autoimmunity-related genes in melanoma. Cancers.

[41] Giampietri C., Scatozza F., Crecca E., Vigiano Benedetti V., Natali P.G., Facchiano A. (2022). Analysis of gene expression levels and their impact on survival in 31 cancer-types patients identifies novel prognostic markers and suggests unexplored immunotherapy treatment options in a wide range of malignancies. J. Transl. Med..

