# Supplementary material for: Schwann Cell Expression Pattern in Human Melanomas: In Silico and Immunohistochemical Analyses
Source: Int J Mol Sci. 2026 Sep 16;27(18):8260. doi: 10.3390/ijms27188260 (PMC13607390; doi:10.3390/ijms27188260)
Supplement: Supplementary file 1 [file ijms-27-08260-s001.zip › ijms-4502532-supplementary.pdf]

Supplementary Table S1

The 100 randomly chosen profiles are reported, with the indication of the HR and logrank p computed for each profile.

Perm n.	HR	Logrank	Profile
1	0.89	P=0.41	TP53, BRCA1, BRCA2, EGFR, APOE, TNF, IL6, VEGFA, MYC, PTEN, AKT1, MTOR, KRAS, NRAS, BRAF, MAPK1, MAPK3, CDKN1A, CDK2, CDK4, CCND1, RB1, SMAD4, TGFBR2, HIF1A, SOD1, CFTR, PNPLA6, ABHD14B, ACAA1
2	0.71	P=0.017	ABL1, ATM, ATR, CHEK1, CHEK2, MDM2, MDM4, FOXP3, STAT3, STAT5A, JAK1, JAK2, PIK3CA, PIK3R1, GSK3B, FOS, JUN, NFKB1, RELA, CXCL8, CXCR4, ITGA2B, ITGB3 , VWF , FBN1 , LMNA , COL1A1, ACOT13, ACTR6, ADCK2
3	1.29	P=0.16	ADIPOQ, LEP, PPARG, SREBF1, FASN, ACACA, CPT1A, MC4R, GCK, HK2, PFKM, PKM, LDHA, ENO1, PDHA1, CS, IDH3A, SDHA, UQCRC1, COX4I1, ATP5F1A, NDUFS1, SIRT1, SIRT3, FOXO1, ZNF703, LRRK2, ADIPOR2, PANK2, AFAP1L2
4	1.41	P=0.039	PARK7, SCN1A, CACNA1C, GRIN2B, DRD2, SLC6A4, MAOA, COMT, OPRM1, OPA3, NGF, APP, PSEN1, MAPT, SNCA, TREM2, CLU, PICALM, BIN1, ABCA7, CR1, PLCG1, PRNP, RYR2, KCNQ1, AR, ESR1, AGBL5, AHCYL2, AKAP7
5	0.53	P=0.0012	ESR2, PGR, NR3C1, NR3C2, RXRA, RARA, THRB, VDR, CYP19A1, CYP17A1, CYP11A1, HSD3B1, HSD17B1, SHBG, KISS1, KISS1R, GNRH1, GNRHR, LHCGR, FSHR, INHA, INHBA, AMH, AMHR2, STAR, NR5A1, DMRT1, ALDH18A1, ALG2, ANKRD9
6	1.27	P=0.28	ASXL1, DNMT3A, TET2, EZH2, SUZ12, ARID1A, SMARCA4, KMT2D, KDM6A, BCOR, SETD2, NSD1, PHF6, CREBBP, EP300, HDAC1, HDAC2, SIRT6, MECOM, WT1, RUNX1, GATA2, ETV6, TAL1, LMO2, IKZF1, BCL11A, AP1M2, APBB3, ARHGAP5
7	0.61	P=0044	DICER1, DGCR8, XPO5, AGO2, EIF2AK2, OAS1, OAS2, MX1, MX2, IFIH1, DDX58, IRF3, IRF7, IRF9, IFIT1, IFIT2, IFIT3, ISG15, USP18, TRIM25, TRIM28, BST2, SAMHD1, ZBP1, ADAR, APOBEC3G, RNASEL, ARL13B, ASB9, ATG4D
8	0.8	P=0.23	SLC2A1, SLC7A5, SLC1A5, SLC25A5, ABCB1, ABCG2, ABCC1, TFRC, FTH1, FTL, IREB2, HMOX1, KEAP1, NFE2L2, SQSTM1, ATG5, ATG7, BECN1, ULK1, RBX1, CUL1, SKP1, FBXW7, CDC25A, CDC25B, WEE1, PLK1, ATP5PB, ATP6V0E1, B3GNT2
9	0.55	P=0.00029	ACVR1, BMP4, BMPR2, SMAD1, SMAD5, SMAD9, NOD2, TLR3, TLR7, TLR9, MYD88, IRAK4, TRAF6, CARD9, CLEC7A, FCGR3A, LILRB1, HLA-A, HLA-B, HLA-C, TAP1, TAP2, PSMB8, PSMB9, ERAP1, ERAP2, CIITA, BBS10, BCAP29, BCL7C
10	1.41	P=0.052	AHSG, SERPINA1, ORM1, HP, TF, CP, FGG, FGA, FGB, PLG, KNG1, PROC, PROS1, THBD, ANXA1, ANXA2, LGALS3, LGALS9, SPP1, CLEC3B, VCAN, BGN, DCN, LUM, COL3A1, COL5A1, COL6A1, BET1L, BEX5, BICD2
11	0.73	P=0.04	ABHD5, ACSL1, ACSL4, ADAM10, ADAM17, ADRB1, ADRB2, AGPAT2, ALDH1A1, ALDH2, ANKRD1, AP1M1, ARF6, ATF3, ATP1A1, ATP2B1,

			AXL, BAX, BCL2L1, BIRC5, C1QA, C1QB, C1QC, CALM1, CALR, CASP3, CASP7, NIPSNAP3A, C1orf35, C2CD3
**12**	**0.58**	**P=0.24**	CBL, CCL2, CCL5, CCR5, CD14, CD36, CD44, CD68, CD86, CLDN5, CTNNB1, CXCL10, CXCL12, DAB2, DUSP1, DUSP6, EEF2, ELN, ENG, ETS1, FABP4, FBLN1, FN1, FOSL1, GJA1, GLUL, HES1, CABP5, CALHM3, CAMK2N1
**13**	**1.3**	**P=0.12**	ITGAV, JUNB, KLF4, KLF6, LGALS1, LPL, MCL1, MET, MFGE8, MRC1, NAMPT, NCAM1, NOTCH1, PDK1, PECAM1, PLAUR, PPARA, PRKACA, PTGS2, RECK, RGS2, SERPINE1, SNAI1, SPARC, SREBF2, TAGLN, TEK, CCDC102A, CCDC146, CDK5RAP1
**14**	**1.18**	**P=0.24**	ABCA1, ABHD12, ACOX1, ADCY3, ADGRG1, ADSL, AFF1, AGTR1, AHR, AKR1C3, ALG13, AMOT, ANGPT1, ANK2, ANLN, APAF1, APLP1, ARHGAP1, ARHGEF2, ASAH1, ATAD2, ATP6V1A, ATP8B1, AVPR1A, B4GALT1, BAG3, BBS1, CEP85, CHCHD2, CHST10
**15**	**0.79**	**P=0.13**	BUB1, CALD1, CAPN1, CAPZA1, CARD11, CBX3, CDC42EP1, CDH2, CDH5, CHUK, CKB, CLIC1, CLTC, COPB1, COQ8A, CORO1A, CRADD, CSF1R, CTPS1, CYBA, CYCS, DAXX, DCXR, DENND1A, DIABLO, DNAJC3, DPYD, CLIC3, CNPY4, COA1
**16**	**1.64**	**P =0.0025**	ECHS1, EEF1A1, ELAVL1, ENO2, EPHB4, ERCC1, ETFDH, FADD, FAM20C, FLOT1, FRS2, G6PD, GADD45A, GNAI2, GNB2, GPD1, GRPEL1, GTF2I, HADHA, HCLS1, HNRNPA1, HSPD1, IFT88, IGF2BP1, IL17RA, IMPDH2, INPP5D, COG1, COQ3, CPNE5
**17**	**0.73**	**P=0.044**	ABCC4, ABHD17A, ACBD5, ACER3, ACTN4, ADAMTS1, ADAMTS5, ADGRL2, AGAP2, AGXT, AHI1, AKAP1, ALDH3A1, ALG3, ANKFY1, ANKRD11, ANP32A, AP2M1, ARL8B, ASNS, ATG12, ATP6AP2, ATP9A, BCL6, BMF, BRD2, BRD7, CRTC3, CSNK1A1L, CTDSP2
**18**	**1.33**	**P=0.043**	CARS2, CCDC88A, CD151, CDK5R1, CHD1, CHMP2B, CKAP4, CLDN1, COG5 , COPZ1, COX6A1, CRELD2, CRYAB, CTDSP1, CTSZ, CYB5R3, DCTN1, DENR, DERL1, DHCR24, DNAJB1, DOCK7, DPF2, EBP, EDC4, EED, EIF4EBP1, CYB5A, DCAF11, DCTN2
**19**	**1.37**	**P=0.037**	EMC1, ENO3, ERLIN1, EXOSC5, FAM107A, FCHO2, FKBP4, FLNB, FUT8, GABARAPL1, GDI2, GMPS, GORASP2, GPX4, GSR, HADHB, HAX1, HINT1, HNRNPC, HSPE1, IFT57, ILVBL, KDELR2, LAMP2, LMAN1, LSM7, MIEF1, DDX23, DENND3, DHRS4
**20**	**1.18**	**P=0.24**	MRPL12, NAA10, NDUFA9, NME1, NPC1, OSBPL1A, PEX13, PHB, PRDX5, RAB7A, SLC30A1, AARS2, ABR, ACAD9, ACOT7, ACTR2, ADNP, AIFM1, AKIP1, ALG1, AMER1, ANAPC1, ANKLE2, AP1S1, APEX2, ARMC10, ARPC3, DNAJC21, DOCK4, NDUFB9
**21**	**1.58**	**P=0.017**	ASPM, ATG9A, ATP5MC3, BAZ1B, BCKDK, BLOC1S1, BRAT1, C1orf112, CANT1, CCT2, CDC37, CDIPT, CEP164, CHMP4B, CITED2, CLPB, COA7, COG2, COX7A2, CPSF6, CRTAP, CSNK2A2, CYFIP1, DCAF7, DDX24, DHX15, DNAJC19, DRG2, MRPL44, MFSD8
**22**	**1.45**	**P=0.021**	ELP3, EMC8, ERCC4, ESCO2, EXOSC3, FAM50A, FASTKD2, FBXO7, FDXR, FIS1, FPGS, FTSJ1, GALK1, GNB5, GORAB, GOSR2, GTPBP3, HCCS, HEATR5B, HIKESHI, HSD17B10, IFT140, IMMP2L, ISCA2, KIAA0586, KPNB1, LAMTOR2, EEF1G, EIF3L, ELMOD3
**23**	**0.69**	**P=0.047**	LONP1, LRPPRC, LYRM7, MCCC1, MPHOSPH10, MRPS22, NAXD, NIPBL, NSMCE3, OPA3, PAXX, PCYT2, PDHX, POLDIP2, PPP1R15B, QRSL1, RARS2, RBM7, SCO1, SDHAF2, SERAC1, TIMMDC1, AATF, ABHD13, ACAA2, LYRM4, MCEE, MED17, AAGAB, ABHD6

**24**	**1.26**	**P=0.13**	AAMP, ABTB2, ACAP1, ACOX3, ADAM23, ADGRE5, AGFG1, AHCYL1, AKAP12, ALDH7A1, ALG9, ANKRD13D, AP1B1, APOBEC2, ARHGAP24, ARPC1B, ASB2, ATAD1, ATP6V1E1, BAG1, BBS9, BCL10, BICD1, BMS1, BOD1, C1orf21, C2orf69, ENOX2, ERCC5, ETFA
**25**	**1.57**	**P=0.0017**	CABIN1, CALU, CAPS2, CCDC6, CCDC85B, CD59, CDC26, CDV3, CEP63, CHMP1A, CISD1, CLDN12, CNBP, COG6, COX11, CPNE3, CRNKL1, CSGALNACT1, CTSC, CYB561, DAD1, DCTPP1, DDX21, DENND4C, DLD, DNAJC10, DPH5, EXOSC2, FAM118A, ACAD9
**26**	**1.7**	**P=0.00029**	DYRK1B, ECH1, EIF2S2, EMC6, ENDOU, EPG5, ERLEC1, EXOSC7, FAM98A, FBXO3, FDPS, FKBP1A, FPGT, G3BP2, GANAB, GNE, GOLPH3, GPX7, GTF3C2, HADH, HNRNPD, HSD17B12, IFT20, INTS6, KDELR1, LARP4, LMF1, FBXO11, FDX2, BBS7
**27**	**0.64**	**P=0.0078**	LRRC8A, MAPRE1, MCM10, MED15, MFSD2A, MIA3, MRPL44, NAGLU, NDUFAF6, NNT, OSBPL8, PCYOX1, PFKP, PNPLA6, AAK1, ABHD16A, ACADM, ACOT9, ACTR1A, ADCK1, ADPGK, AFG3L2, AGMAT, AHNAK, AKIRIN2, ALAD, ALDH9A1, GALK2, GINS4, BCAP31
**28**	**1.75**	**P=0.0061**	ALG14, AMFR, ANXA11, AP2S1, APOLD1, ARFGAP1, ARID5A, ASAH2, ATP1B3, ATP5ME, ATP6V1G, BBS12, BCL2L13, BLOC1S2, BORCS7, C12orf57, CACYBP, CALCRL, CAMK1D, CAPNS1, CCDC47, CCNB2, CDAN1, CDC123, CDIP1, CEP70, CHCHD10, GLRX2, GNPTAB, BEND6
**29**	**1.35**	**P=0.039**	CHST14, CLIC4, CMTM6, COA3, COG7, COX18, CPVL, CRACR2A, CSDE1, CUTA, CYB5B, DCAF13, ERCC6L2, ETHE1, EXOSC10, FAM114A2, FAM171A1, FCHO1, FKBP15, FUZ, GALT, GOLIM4, GPATCH8, GSTM3, HACD2, HBS1L, HEBP1, GPATCH2, BRD9, CALHM6
**30**	**0.81**	**P=0.17**	HSPB11, IFT43, IMP3, INTS14, KATNB1, LAMB2, LDHD, LETM1, LMBR1, MAN2B2, MAPKAPK3, MCOLN1, MED27, MFN2, MPC1, MRPS34, AASDH, ABHD10, ACER2, ACSL5, ACTL6A, ADAM9, ADCK5, ADRM1, AEBP1, AFDN, AGPAT5, HIGD1B, HNRNPU, IFT74
**31**	**0.55**	**P=0.0059**	AHCTF1, AKAP10, ALDH6A1, ALG10B, AMPD2, ANKRD17, AP1AR, APPL2, ARHGAP21, ARL6IP1, ASXL2, ATAD3A, ATP5MG, ATP6V1C1, B3GNT5, BAG6, BAZ2A, BCAP31, BCL2L12, BET1, BEX2, BIRC6, C11orf24, C1QTNF6, C7orf50, CAB39L, CALR3, IMPA1, INTS4, CAMK2D
**32**	**0.7**	**P=0.017**	CCDC93, CCNB1IP1, CDK12, CEP57, CHCHD3, CHST3, CLN6, CNPY3, COA6, COG4, COQ5, CPD, CREG1, CSNK1G3, CUX1, CYB5D1, DAGLB, DBN1, DDX41, DENND2D, DHX29, DNAJC11, DOCK6, DPH6, DRAP1, DYNC1H1, ECHDC2, LMBR1L, CCDC170, CEP104
**33**	**1.85**	**P=0.0000055**	EEF1E1, EGLN1, EIF3H, ELP4, EMC7, EPHX2, ERLIN2, EXOSC9, FBXO8, FDX1, G6PC3, GLOD4, GNPDA1, GOLT1B, GPATCH11, GRINA, HINT2, HSD17B4, IFT27, ILF2, INTS12, KCTD5, KIAA0753, LPCAT4, LYRM4, MCM7, MED20, LYRM1, MCM3AP, MED18
**34**	**1.18**	**P=0.32**	MFSD8, MRPL52, NDUFAF5, AARSD1, ABHD11, ACBD6, ACOX2, ACTR10, BRF2, C1orf94, C2CD4B, CABP7, CALHM2, CAMK1G, CCDC120, CCDC138, CDK5RAP2, CEP120, CHCHD6, CHPF2, CLHC1, CNPY2, COA4, COG8, COQ7, CPNE8, CRYBG3, MFSD10, MRPL27, CHCHD10
**35**	**0.82**	**P=0.23**	CSNK2B, CTDP1, CYB5RL, DCAF5, DCTN6, DDX27, DENND1B, DHRS13, DNAJC16, DOCK8, DPH2, DTWD1, DYNC1I1, EEF1B2, EIF3K, ELMOD1, ENOX1, ERCC8, ETNK2, EXOSC1, FAM104A, FAM162A, FBXO22, FLAD1, GINS1, GLRX3, GNPAT, NDUFB7, AASDHPPT, ABHD18
**36**	**1.44**	**P=0.045**	ACOT11, ACTR1B, ADIRF, AIFM2, AKAP2, ALDH4A1, ALG5, AP1S3, ARHGAP18, ARMCX1, ASB11, ATP5MC2, ATP6V1F, B3GALT6, BBS5,

			BCAR1, BEND5, BRIX1, C11orf58, C1orf43, CABP4, CALHM1, CAMK1, CCDC141, CHST12, CLCC1, CNPY3, COA7, COG6, COQ8A
**37**	**1.66**	**P= 0.0018**	CDK5RAP3, CEP152, CHCHD7, CNPY1, COA5, COG3, COQ9, CPNE9, CSNK1E, CYB5R1, DCAF10, DDX49, DENND4A, DHRS3, DNAJC7, DOCK9, DPH3, DTWD2, DYNC2H1, ECHDC1, EIF3M, CPNE4, CRNKL1, CSNK2A2, CTDSP2, CYB5B, DCAF4, DCTN4, DDX52, DENND5A
**38**	**1.65**	**P=0.0069**	EXOSC4, FAM167A, FBXO16, GINS3, GNPNAT1, GPATCH1, HIGD2B, HNRNPL, IFT172, INTS10, LMBRD2, LYRM9, MCM8, MED22, MFSD12, MRPL58, DHRS7B, DNAJB14, DOCK10, DPH5, DRAM2, DYNC1LI2, ECHDC2, EEF1AKMT3, EIF3E, ELMOD2, ERCC3, ETNK1, EXOSC6, FAM117A
**39**	**0.63**	**P= 0.0098**	NDUFB6, NIT1, PANK4, PNPLA7, AAGAB, ABHD6, ACAD9, ACOT9, ACTR8, ADCK1, ADORA3, AIMP1, AKIRIN1, ALDH3A2, ALG10, ANKRD13C, AP1AR, ARHGAP21, ARL8A, ASB15, ATP5ME, ATP6V1C2, B3GAT2, BBS7, BCAP31, FAM161B, FBXO45, FDX1, GALT, GINS2
**40**	**1.51**	**P= 0.023**	BEND6, BRD9, C1orf56, C2orf69, CABP2, CALHM6, CAMK2D, CCDC115, CCDC170, CEP104, CHCHD10, CHST12, CLCC1, CNPY3, COA7, COG6, COQ8A, CPNE4, CRNKL1, CSNK2A2, CTDSP2, CYB5B, DCAF4, DCTN4, DDX52, DENND5A, DHRS7B, DNAJB14, GNPTG, GOSR2
**41**	**0.7**	**P= 0.024**	AAMDC, ABHD12B, ACAP3, ACSF3, ACTL10, ADAMTSL4, ADCY10, ADD3, AFF4, AGFG2, AHNAK2, AKAP11, ALKBH7, AMMECR1L, ANKRD36C, AP4M1, ARFGEF3, ARHGAP32, ARID4B, ASCC2, ATAD3B, ATP11A, ATP6V1E2, B4GALT7, BAG4, BBS12, BICDL2, BLVRA, BORCS7, C15orf40
**42**	**1.46**	**P=0.011**	C19orf53, C2CD6, CABLES2, CALML6, CAPN10, CCDC47, CCDC61, CDK10, CEP128, CHMP4B, CHPF, CKAP2L, CLN6, CNBP, COA3, COLEC11, COPRS, COPS7A, CRADD, CSGALNACT2, CTNND1, CWC15, DARS2, DCBLD2, DHTKD1, DNAJB2, DPYSL5, DSTYK, EBPL, ECE2
**43**	**1.43**	**P=0.01**	EIF4ENIF1, EMC8, ENO4, EPG5, ERLIN2, FAM120C, FAM149B1, FBXO34, FKBP10, FZD4, GATAD2A, GIPC3, GLT8D2, GNAI3, GPR137B, GRPEL2, HADHA, HAX1, HERC6, HSD17B13, IFT43, ILF3, INO80E, ISOC1, KIAA0753, KRTCAP2, LMF2, LRRC40, MAPKAPK5, MCOLN3
**44**	**0.81**	**P=0.18**	ABHD17C, ACBD7, ACOT7, ACTR5, ADAMTSL5, ADCY7, ADGRL4, AGPAT5, AHI1, AKAP12, ALDH1L2, AMOTL1, ANKRD39, AP3M2, ARFIP2, ARHGAP24, ARMCX6, ASXL2, ATG2A, ATP8B2, ATP9A, B4GALT6, BAZ1B, BBS1, BCL10, BIRC6, BRPF3, LARP6, LMOD3, LRRC8D
**45**	**1.82**	**P=0.00027**	C1QBP, C2orf81, C5orf22, CALCOCO2, CAPZA3, CCDC28A, CDCA4, CEP44, CHMP2A, CHST6, CLCN7, CNNM4, COA6, COG4, COLGALT1, CORO7, CPNE3, CRTC2, CSNK1G3, CTBP1, CUTA, DAB2IP, DCAF13, DCTN3, DDX54, DENND6A, DHX29, DNAJC11, DOCK7, MAPRE3
**46**	**1.31**	**P=0.18**	DPH1, DSG2, ECHDC3, EEF2K, EIF4G2, ELP4, ENO3, ERCC6L2, ETAA1, EXOC3L2, FAM114A2, FAM177A1, FBXO38, FNDC3B, GATAD1, GIPC1, GNB5, GPR176, HADHB, HECTD2, HMGXB4, HNRNPDL, IFT122, IKZF5, INTS8, IQCB1, KDM7A, KIAA0408, MINDY2, MRPL52
**47**	**0.73**	**P=0.056**	AAK1, ABCA9, ABHD8, ACBD5, ACOT4, ACTN1, ADAMTS10, ADCK5, ADGRG6, AEBP2, AGAP3, AHSA1, AKAP13, ALDH1A3, AMFR, ANKRD44, AP3B2, ARFGAP1, ARHGAP30, ARMC10, ATG9A, ATP1B3, ATP6AP1L, ATP8A2, BAIAP2L2, BBS2, BCL2L13, BICRA, BUB3, C10orf88
**48**	**1.26**	**P=0.12**	C1orf112, C3orf33, CAB39L, CALCOCO1, CAMK1D, CAPN13, CCDC12, CCDC85B, CDK12, CEP76, CHMP1B, CHST11, CLINT1, CPNE2, CRELD2,

			CSNK1D, CTPS2, CWC22, DCP1B, DCTN5, DDX31, DENND2A, DHX37, DNAJC5B, DOCK6, OSBPL3, NUP50, MRPL41, NAA35, NDUFB11
**49**	**0.85**	**P=0.37**	AAMP, ABCB8, ABHD2, ACAT2, ACSL5, ACTR3B, ADAT2, ADGRE2, ADIPOR1, AFMID, AGMAT, AHCY, AKAP10, ALDH6A1, AMN1, ANAPC13, ANKRD17, AP1B1, ARF4, ARHGAP10, ARL6IP1, ASNSD1, ATF7IP, ATP2C1, ATP5F1D, ATP6V0D1, BAG2, BCL2L12, BID, BOLA3
**50**	**1.58**	**P= 0.0018**	C1orf21, C15orf48, CABIN1, CALU, CAPNS1, CCDC93, CDK14, CEP19, CHMP5, CHSY1, CLDN12, CLPX, COASY, COQ4, CPD, CREG1, CSNK2A1, CTNNBL1, CYB5R4, DCTPP1, DDIT4, DECR1, DNAJC9, DPH6, DYRK3, ECHS1, EEF2, EIF2S2, EMC1, ENO2
**51**	**0.86**	**P=0.43**	MAP3K6, MEAF6, MECR, MFAP2, MIB2, MIER1, MIGA1, MFN2, MMEL1, MTFR1L, MTHFR, MUL1, MUTYH, NBPF3, NGF, NOL9, NRAS, NOTCH2, OLFML3, OMA1, OVGP1, PARK7, PINK1, PLOD1, PRAMEF10, PRMT6, PRXL2B, PSRC1, RAD54L, RAP1A
**52**	**1.57**	**P=0.0056**	RBM15, RCC2, REG4, RHBDL2, RIMS3, RLF, RNF11, RNF19B, RNF220, RPA2, RSPO1, S100A1, SAMD11, SDC3, SFPQ, SGIP1, SH3BGRL3, SLC16A1, SLC2A1, SPSB1, STIL, SYCP1, SZT2, TACSTD2, TAL1, TGFBR3, THRAP3, TIE1, TM2D1, TMCO2
**53**	**1.17**	**P=0.33**	TMCO4, TMEM50A, TMEM59, TMEM69, TMEM201, TMEM222, TOE1, TRABD2B, TRAPPC3, TRIT1, TSHB, TTC39A, UBR4, UROD, USP1, USP48, VAV3, VPS13D, VTCN1, WARS2, WDR77, YBX1, ZCCHC17, ZFP69, ZMYM1, ZNF436, ZNF684, ZYG11B, A1BG, A2M
**54**	**1.46**	**P=0.031**	BRCA2, C3, CASP3, CAT, CCL2, CD4, CDK2, COL1A1, CRP, CTNNB1, CXCL12, CYP1A1, DMD, EGFR, EPAS1, ESR1, F2, F5, FASN, FBN1, FGF2, FOXP2, FTO, GAPDH, GDF15, GNAI2, GPI, GSTP1, ALDH1B1, ALDH2, ALDH3A1
**55**	**1.65**	**P=0.26**	BEST3, BEST4, BET1, BET1P1, BEX1, BEX2, BEX3, BEX4, BEX5, BFSP1, BFSP2, BGLAP, BGN, BHLHA9, BHLHA15, BHMT2, BICC1, BICD1, BICRA, BICRAL, BID, BIK, BIN1, BIN2, CENPVL2, BIN3, ALDH1B1, CD3D
**56**	**0.56**	**P = 0.00037**	ABCG2, SLC5A3, CFLAR, HLA-DMA, ALOX15, CCL17, NEUROD2, MAP3K13, E2F3, SMARCE1, NUMB, UGT1A4, HSF4, EPHB4, FGF21, IRF4, EPHA5, KAT6B, BCL11B, BIRC3, CDH3, SETD2, MAP3K8, RPS6KA2, KITLG, IL1RL2, TNFRSF25, POU6F1, IL18, LEF1
**57**	**0.7**	**P=0.36**	PRKCZ, CYP7B1, SLC6A8, KLF15, MAPK14, PRDM8, TNFRSF18, IL23R, WNT10B, RPS6KA1, SLC4A1, DNAJB3, CSNK2A2, CDKN1C, DNAJC9, SULT1E1, ACTL6B, TICAM2, MAP2K3, IL13RA1, KDM2A, SLC3A1, CYP26B1, CCL13, TNFRSF8, SETD1A, RPS6KB1, KLF8, CHD4, SP2
**58**	**1.8**	**P= 0.15**	SETD1A, RPS6KB1, KLF8, CHD4, SP2, CYP2C19, E2F1, KLF3, SFRP4, SETD4, HSPA4, TGFB2, TNFRSF19, MFNG, TNFSF13B, JAG2, HFE, SOX14, DNAJC6, SMAD4, PYHIN1, ATF3, CDH6, EP300, MLST8, RSPO4, FOXN2, BRCA2, NRGN, FANCB
**59**	**0.8**	**P=0.16**	IRAK4, SMARCA1, EHMT2, PDK2, POU6F2, PRDM16, NLRP13, JAG1, DSG2, FOSL1, FKBP7, SETD7, CYP3A4, IL1RL1, DSC3, PDK1, PAX4, CREB1, MAPK12, SOX6, MAP2K1, IL22RA2, EPHA1, TLR6, IL22RA1, SRCAP, PRKAB1, RPS6KC1, IL15RA, BRIP1
**60**	**0.6**	**P= 0.22**	SMARCA2, KLF5, TNFRSF6B, SOX21, IL13, NLRP8, PRMT6, RELB, IL26, TGFBR1, PRKD3, PAX3, HLA-DRB4, PTGS2, ABCC8, CXCL10, TP73, RIPK1, SLC3A2, FBXO21, FOXA1, DNAJB2, CSNK2A1, XRCC1, APCDD1, CHD6, SULT1A3, IL25, ERCC6, MAP3K9

**61**	**1.39**	**P= 0.03**	AKT2, ALK, APC, ARID2, BAP1, BARD1, BLM, CDH1, CDK6, CDKN2A, CTNNB1, ERBB2, FANCA, FGFR1, FLT3, GNAS, HNF1A, HRAS, IDH1, JAK3, KIT, MLH1, MSH2, MSH6, NF1, PALB2, PDGFRA, PIK3CB, PMS2, POLE
**62**	**1.14**	**P= 0.35**	PPARG, RAD51, RET, SDHB, SMAD2, TSC1, ABL2, AR, BCL2, BCR, CCND2, CDKN2B, CTLA4, CYP2D6, DDB2, E2F1, ERBB3, ERCC2, FANCC, FANCD2, FGFR2, FGFR3, GALT, GSTM1, HPRT1, IDH2, IL2, IRF1, MDM1, MET
**63**	**0.48**	**P=0.081**	NQO1, PDGFB, POLD1, STAT1, AMPD1, APAF1, BAX, BCL6, BMP2, CASP8, CASP9, CCNE1, CD19, CDKN1B, CHUK, COL2A1, CSF1, CTLA4, CYP1A2, CYP3A5, DDX5, E2F2, ERBB4, FANCF, FANCG, FLT1, GATA3, GJB2, GSTT1, HDAC3
**64**	**0.8**	**P= 0.14**	HNF4A, HOXB13, IL10, IRF6, JAK3, LRP5, MYB, NF2, ABL2, ADAM17, AKT3, ARID1B, BCL2A1, BCL7A, CDH13, CDK1, CDK8, CDKN1C, CHD2, CHST1, COL1A2, COL4A1, CSF2, CTNND1, CYP2C9, CYP3A4, DDB1, E2F3, ERBB4, FANCA
**65**	**0.63**	**P=0.0049**	FANCD2, FLT4, GATA4, GJB1, GSTP1, HDAC4, HNF1B, HOXC13, IL12A, IRF8, KDR, LRP1, MYC, NFKB2, AURKA, AXIN1, BCL3, BMPR1A, BTK, CBLB, CCND3, CD79A, CDKN2C, CHFR, COL10A1, CSF3, CTNNA1, CYP2A6, CYP2C19, DDX3X
**66**	**0.82**	**P= 0.17**	E2F4, FANCB, FANCL, GATA6, GJB3, GSTA1, HDAC5, HOXD13, IL13, IRF2, LRP2, MYCL, NFKBIA, NOTCH2, ABL1, BTK, CARD11, CCND3, CDK1, CDK4, CDK6, CDK8, CDKN2B, CHD8, CIC, CREBBP, CSF1R, CTNNB1, CYLD, DDR2
**67**	**0.56**	**P=0.0066**	DIS3, EED, ELF3, EP300, ERBB3, ERBB4, EZH2, FANCA, FANCD2, FANCG, BAP1, BCOR, BCORL1, BRAF, BRIP1, BTG1, CALR, CASP8, CASZ1, CDH1, CDKN1B, CDKN2C, CEP57, CHEK1, CHEK2, CIC, COL1A1, CREB3L1, CSF3R, CTNND1
**68**	**0.68**	**P=0.0083**	CYLD, DAXX, DDX41, DNM2, DNMT3A, DOT1L, EGFR, ELF3, EML4, EPHA3, ERCC2, ERCC4, ESRRA, ETV1, ETV6, A4GNT, AAAS, AACS, AAGAB, AAK1, AAMDC, AARSD1, ABAT, ABCA1, ABCB4, ABCC2, ABHD1, ABI1, ACADL, ACAT1
**69**	**1.6**	**P=0.0039**	CD47, BACE2, P2RY10, IGF1, OTUD6A, BLCAP, NDUFAB1, RAD23B, P2RY14, NBR1, RALB, NBPF10, NDUFA2, AKAP8, L1CAM, PARP15, ORC1, LEP, IL10, ICAM3, RAB14, SALL3, OLFM4, CASP1, LIF, GPD1, AGXT2, LAMC2, CD44, EEF1A1
**70**	**0.55**	**P=0.00021**	ADH7, EPHB1, ORC5, ADSL, RAB30, AHI1, BEAN1, DNALI1, FOXI1, ABHD2, JAZF1, AHNAK, NANOS3, KRAS, SAMD8, IL11, NAP1L2, FGR, LNX2, OXTR, F7, HBD, RABL3, DSC1, DHH, BDKRB1, LINGO1, RAB6B, RAB31, PAEP
**71**	**0.69**	**P= 0.017**	PANK4, BEGAIN, KHSRP, ENPP1, ADH1B, KAT6A, LMO4, GGCX, F12, AGFG1, HDAC5, DYNC1H1, RALGPS1, IDO1, RAB36, CDK7, OXT, RAF1, ADCY9, RAB33A, SAV1, OTUD1, ELF1, P2RY8, JAG1, KAT2A, ERBB2, IGHM, GNAS, KRT1
**72**	**1.77**	**P= 0.0048**	RAB10, FOXA2, AHNAK2, NAGLU, LAT, EIF4A1, P4HA2, FMO1, ADAM10, RAB12, BATF, KRT7, CD4, OSBPL9, CDKN3, OTUD4, BAK1, LRP5, CCNA1, P2RY12, LAMB1, HBA2, KRT6A, F13A1, LHX1, GLRA1, MAPK1, HBG1, DLL1
**73**	**1.6**	**P=0.0091**	ERG, RAB7A, QSOX1, BCL2A1, NCAM1, PALB2, LPL, MEF2B, ADK, RAG1, P2RY1, OTUB2, RAB5A, MCM6, ICE2, HDAC8, RAB35, LGMN,

			CBX1, HEXB, RAD54B, MAOA, EMCN, AKR1C4, KEL, RAB1B, QSOX2, RAB38, EPAS1, EHD1
**74**	**1.31**	**P=0.12**	DRD2, BCL11A, GAN, ERBB4, NANOS1, LOX, ORC3, RAD51C, HIBCH, CALB1, HBM, PANK1, KIF11, FGG, BARD1, NDUFA7, HK3, KRT6B, ETFA, DLL4, F13B, SAT2, M6PR, ADIPOR1, HES5, ADAT3, OLFM3, IAPP, ADAM15, DTNA
**75**	**1.35**	**P=0.13**	LOXL1, DDB1, FOXD1, ADARB1, GLB1, RABL2A, ELAVL2, MDH2, BARX1, OTUB1, ENG, GJB2, HBP1, HDLBP, AGBL1, RAB25, ID3, QPCTL, ID4, CCND3, SAMD1, SCGB3A2, LINS1, PARP4, NCAN, LIPN, NDUFS2, DGAT2, MALT1, RAB32
**76**	**1.43**	**P=0.036**	RAB37, GRM1, IFI44, KCNQ1, HINT1, AK2, KRT17, GJB6, NDUFA4, DPEP1, GOLGA2, EMP1, RAD21, MASP1, F9, RAB3C, GALT, ABCA7, IFT74, GDF15, OXCT1, IFI35, RAB1A, LIMA1, LMO2, MATN1, CAV1, RABEP2, BBS4, DRD1
**77**	**0.63**	**P=0.0075**	P3H1, NAPEPLD, ABHD5, MCHR2, IL15, KLF5, PAK2, DOK4, IBA57, MFAP3, BIRC3, RAB2A, ACTR3, CD3G, NDUFA1, NDUFA3, RAB9B, BCL6, HAMP, SAFB2, KLF1, NCAM2, GRHPR, METTL2A, OVGP1, NAT2, SCN3A, CD247, CD3E, FOXH1
**78**	**1.26**	**P=0.17**	MC5R, FGA, KAT6B, MEIS1, RAB13, RAB39B, RAD9A, GBE1, PAPOLA, P2RX4, MCM3, SCN2A, CCDC50, AKR1C1, AKR1C2, BCL7A, GAMT, AK1, OSBPL2, RAB8B, GNS, AK4, MAN2A1, GFAP, KIF1B, LAMC3, NANOG, PAX2, BIVM, BDKRB2
**79**	**0.66**	**P=0.0033**	AKAP9, AHDC1, FHL1, RAD17, MATN3, NCK1, SAMD9, AFF3, NDP, HK1, LIPC, LAMP3, P2RY13, P2RX5, MAPK3, NCCRP1, GDAP1, KDR, OCLN, RACGAP1, NDUFA9, CD79B, RAB20, PARP3, JUNB, FOXC2, LAMP2, LGALS3, NAT1, ADCY5
**80**	**0.74**	**P=0.094**	P2RY6, HES4, CAV2, ICOS, OCRL, ABCB11, PARP10, ORC6, MAPK8, KRT81, NDRG1, GMPR, CAPZA2, SCARB1, BCL3, P2RX2, RAB3A, GAPDHS, MEF2D, BCL7C, LRP1, GJA1, BIRC7, RAB24, BHLHE40, P2RX3, MCCC1, IFI44L, PARP1, RAD1
**81**	**0.65**	**P=0.011**	GCK, OGN, LMTK3, GJB3, PARP9, ACADVL, METTL1, RABEPK, GLUD1, NDN, IGFALS, ICOSLG, RAB41, LPIN1, RAB39A, ACTN2, MC3R, RAB8A, OSBPL10, ADAM17, PAX1, PAX3, KRT5, PAN2, MC1R, MFAP5, SCN1A, ACAT2, ADAMTS3, LOXL3
**82**	**0.8**	**P=0.16**	CCNE2, IFI16, BIRC2, IL12B, RAB40B, LHX3, OSBPL7, GIP, CD8A, LPIN2, LGALS1, OSBPL3, SAA4, FHL2, OLR1, CASP2, HFE, GCNT2, RAB5B, ADORA1, PAN3, ORC4, SAMD14, MDH1, ALAS1, DRD5, RAB6A, HBB, AGL
**83**	**1.3**	**P=0.068**	AFF2, KREMEN1, KMT2B, ACBD5, RAB15, MCM4, HES2, GRIA1, RAB22A, AKAP1, LOXL4, RAD54L, RAB11A, RAB26, CD40, OSBPL5, SAA2, SAT1, ID2, BCL7B, RAD23A, HGFAC, OLFM2, NCOR1, ERN1, RABGGTB, RAB40A, FMO4, LHX9, MCM7
**84**	**1.48**	**P=0.015**	GHR, LARS2, FGL2, BACE1, RAB27B, HES1, NDUFA8, ERF, ICAM5, DSP, NAV3, BCORL1, MAPK9, CDK9, CBX2, ADCY1, DSG4, AGPS, NDRG3, LINC00486, QPCT, ACOT1, AGTPBP1, BANK1, P2RY2, RAB21, KMT2C, LAMC1, HGS, SALL2
**85**	**0.75**	**P=0.098**	BIRC8, HCCS, GM2A, OSBPL11, DSC2, LNX1, F3, HK2, SALL4, EPHB2, CAPN2, P2RY4, DOK5, ICMT, IGF2, ADGRV1, RAG2, NBPF20, MFN1, PAPOLB, ADM, KLF2, RABGGTA, NDUFA6, FN1, KIF23, MEF2C, F10, IFT88, AKAP7

**86**	**1.82**	**P=0.00059**	KRT8, DDX17, AARS2, PABPC4, AFG3L2, MCM5, KLF4, RAB27A, HDAC9, LOXL2, ADAM12, DPT, HES3, EPHA1, KIF20A, IGF1R, SAA1, DRD4, HCK, KCNJ11, NCOR2, FMO5, RAB5C, MEF2A, ACVR2A, RABIF, KRT19, CDC42, RAB11B, NDUFA5
**87**	**0.88**	**P=0.45**	FGB, ERBB3, ALAS2, F5, HGF, EPN1, CD79A, NDRG4, NCK2, ABCC3, EMB, RALA, PAK3, NAP1L1, NAV2, AKR1B1, CD3D, MC4R, ENTPD1, GOLT1A, HEXA, PAK4, MCHR1, IFIH1, PARP12, ICAM4, ICAM1, GGH, DKK1, AKR1A1
**88**	**0.77**	**P=0.15**	BIRC6, MATN2, BBS2, GRIA4, ELOVL1, BECN1, IDO2, AEBP1, GCH1, P2RX1, RAB28, CAMK2A, IFI30, QRICH2, OTUD3, ACER1, DOK6, HBA1, HLA-A, CACNB1, RAB3D, ORC2, ERO1A, RAB7B, PANK3, PABPN1, KRT10, RAB4A, ACYP1, SAFB
**89**	**0.68**	**P=0.02**	HAAO, HIPK1, MCM2, GAS2, NDNF, RABL2B, MCCC2, ADCY6, MASP2, AGAP1, SCARB2, ADH5, ENO1, ESRRG, CCDC8, RAMP2, QRICH1, PARP14, GJB1, MAGEA1, DNMT1, PARP2, PAPOLG, ABCD1, ABCC4, PAM, DSG3, AADAC, OGG1, KLF3
**90**	**0.65**	**P=0.032**	HIF1A, OTUD5, RALGPS2, RABEP1, DYNC1I1, AGRN, ID1, ADD1, IL10RA, MC2R, ENO2, ACTB, RAB4B, FMO3, SCEL, OLFM1, HADH, FHL3, PAK6, ELK1, GPT, ADAM8, RAD51D, NDUFS1, ICAM2, NDUFA11, RAD51B, IL10RB, OTUD7A, GALC
**91**	**0.68**	**P=0.0053**	MAN1A1, RAB2B, SCLY, GGT1, DOK2, NDUFA10, PANK2, DSG1, KMT2D, GJB5, RAB9A, RAD18, DOK7, CD28, RAMP1, GAA, LMO1, NDRG2, PAX5, GATAD2A, RAB23, LIFR, NDUFS3, RAB33B, KRT14, EFNA1, SAMD11, ACTG2, OPTN, P2RX7
**92**	**1.72**	**P=0.0011**	SALL1, GBA, IFI6, SART1, LHX2, LAMP1, MFAP2, DES, DMRT1, HIP1, KCNH2, ACTG1, AFF1, HBG2, FOXF2, GFPT1, SART3, F8, BCL10, ACBD3, ADAMTS2, SBDS, MAOB, DRD3, PAK1, AHCY, KMT2A, PABPC1, ERC1, ABLIM1
**93**	**0.4**	**P=0.018**	HES7, PASK, RAB3B, ABCA3, CCNB2, NDC1, NBPF1, MEIS2, FOSB, SAMD12, KAT2B, IL13, OTUD6B, EPB41, NBR2, RAB43, OSBPL1A, METTL3, SAMD13, ADRB3, NAV1, IFI27, RALGDS, FOXE1, LAMP5, BIRC5, RAB17, GCLM, GRIA2, OTUD7B
**94**	**1.63**	**P=0.00644**	LRP2, EPS15, FMO2, F11, KIF1A, KIT, AKAP5, IDH3B, KRIT1, MAPK10, RACK1, CAV3, LMNA, ELOVL2, G6PD, PAFAH1B1, BCL9, SCMH1, P2RX6, EGFR, P4HA1, RAB42, CACNA1A, FOXF1, JAK3, DLL3, DNM1, IGF2R, PAFAH2, FOXC1
**95**	**1.46**	**P=0.33**	PRKCZ, CYP7B1, SLC6A8, KLF15, MAPK14, PRDM8, TNFRSF18, IL23R, WNT10B, RPS6KA1, SLC4A1, DNAJB3, CSNK2A2, CDKN1C, DNAJC9, SULT1E1, ACTL6B, TICAM2, MAP2K3, IL13RA1, KDM2A, SLC3A1, CYP26B1, SP2, CYP2C19, E2F1, KLF3, SFRP4, SETD4, HSPA4
**96**	**0.52**	**P=0.11**	TGFB2, TNFRSF19, MFNG, TNFSF13B, JAG2, HFE, SOX14, DNAJC6, SMAD4, PYHIN1, ATF3, CDH6, EP300, MLST8, RSPO4, FOXN2, BRCA2, NRGN, FANCB, IRAK4, SMARCA1, EHMT2, PDK2, POU6F2, PRDM16, NLRP13, JAG1, DSG2, FOSL1, FKBP7
**97**	**0.83**	**P=0.26**	SETD7, CYP3A4, IL1RL1, DSC3, PDK1, PAX4, CREB1, MAPK12, SOX6, MAP2K1, IL22RA2, EPHA1, TLR6, IL22RA1, SRCAP, PRKAB1, RPS6KC1, IL15RA, BRIP1, SMARCA2, KLF5, TNFRSF6B, SOX21, IL13, NLRP8, PRMT6, RELB, IL26, TGFBR1, PRKD3
**98**	**0.36**	**P=0.007**	PAX3, HLA-DRB4, PTGS2, ABCC8, CXCL10, TP73, RIPK1, SLC3A2, FBXO21, FOXA1, DNAJB2, CSNK2A1, XRCC1, APCDD1, CHD6, SULT1A3,

			IL25, ERCC6, MAP3K9, S100A1, HSP90AA1, BCL3, TIRAP, CXCL14, FOXF1, MAP2K3, PCDHGB1, ACTL8, KLF15, GSTA3
**99**	**0.46**	**P=0.063**	FOSL2, HEY2, FOXD2, GSTM4, IL5RA, PRKAA1, AHSA1, STK11, PDGFRB, EGR2, ABCF1, IL21R, CCL5, HLA-DRB5, JUN, INO80, HES1, SLC6A11, PBRM1, NLRC5, MAPK3, SP9, GSTT2, HES6, FOXS1, CDH23, PAX7, ABCG1, NFATC2, KLF6
**100**	**0.56**	**P= 0.0006**	FKBP5, IRF5, TRAF2, MAP4K5, HES4, MAP3K3, SP8, MST1R, CREM, FOXA2, WNT9B, RELA, PRKD1, NKX6-2, PCDHGA5, MAP2K6, FKBP8, CCL18, IRAK1, FOXB2, CCL3, KLF9, SIRT6, TFR2, SMARCB1, HIF1A, DEPTOR, HAND1, IL31RA, TBX1
